# Development of a radiomics-based model using computed tomography imaging to assess the incidence of extrapulmonary organ involvement in *Mycoplasma pneumoniae* pneumonia and to predict recovery times: a multicenter study

**DOI:** 10.3389/fmed.2025.1732165

**Published:** 2026-01-15

**Authors:** Jiawei Hao, Liyong Zhuo, Shan Gao, Huan Meng, Zijun Song, Xiaoping Yin

**Affiliations:** 1 Department of Radiology, Affiliated Hospital of Hebei University, Baoding, China; 2 Hebei Key Laboratory of Precise Imaging of Inflammation Related Tumors, Baoding, China; 3 Beijing Institute of Brain Disorders, Laboratory of Brain Disorders, Ministry of Science and Technology, Collaborative Innovation Center for Brain Disorders, Capital Medical University, Beijing, China; 4 Department of Critical Care Medicine, Baoding First Central Hospital, Baoding, China

**Keywords:** computed tomography, extrapulmonary organ involvement, *Mycoplasma pneumoniae* pneumonia, predictive analysis, radiomics

## Abstract

**Objectives:**

This study aimed to develop a predictive model, based on radiomics, to assess the occurrence of extrapulmonary organ involvement and predict recovery durations in children with *Mycoplasma pneumoniae* pneumonia (MPP).

**Materials and methods:**

We retrospectively included 556 confirmed MPP patients from three medical centers between October 2022 and December 2024. Feature parameters were selected and weighted using Z-score normalization and LASSO. A logistic regression model was constructed to assess extrapulmonary organ involvement. Model performance was evaluated using the area under the curve (AUC), calibration curves, and decision curves, with comparisons between models conducted using the DeLong test. For predicting recovery duration, a separate model was developed based on selected features and was evaluated using mean squared error (MSE), the coefficient of determination (R^2^), and mean absolute error (MAE).

**Results:**

In the evaluation of the extrapulmonary organ involvement model, the Radiomics Model showed statistically significant differences when compared with both the Clinical Laboratory Model [(AUC = 0.73; 95% CI, 0.53–0.88) vs. (AUC = 0.67; 95% CI, 0.49–0.84), *p* < 0.05] and the Image Feature Model [(AUC = 0.73; 95% CI, 0.53–0.88) vs. (AUC = 0.65; 95% CI, 0.45–0.80), *p* < 0.05]. Significant differences were observed between the Clinical Laboratory Model and the Image Feature Model in the combined organ involvement group (*p* < 0.05), but no statistical difference was found in other groups (*p* > 0.05). The Integrated Model outperformed the Radiomics Model, Clinical Laboratory Model, and Image Feature Model, achieving the highest predictive performance (AUC = 0.94; 95% CI, 0.84–0.99), with all differences being statistically significant (*p* < 0.01). For predicting recovery duration of extrapulmonary organ involvement, the modified MSE was 6.0, the modified MAE was 1.9, and the modified R^2^ Score was 0.6825, indicating acceptable prediction performance.

**Conclusion:**

This study demonstrated that incorporating radiomics significantly improved the predictive accuracy of clinical laboratory parameters and imaging features for assessing extrapulmonary organ involvement and forecasting recovery durations in MPP patients. This approach provided an effective tool to enhance diagnostic efficiency for clinicians.

## Introduction

*Mycoplasma pneumoniae* (MP) is a significant pathogen responsible for community-acquired pneumonia in children, and accounting for approximately 7–30% of all pneumonia cases (1, 2). When the host’s immune system is weakened, MP can develop into *Mycoplasma pneumoniae* pneumonia (MPP). Studies have shown that MP undergoes large-scale, cyclical outbreaks every 3–7 years, with each outbreak lasting from several months to years (3, 4). Given this pattern, early and accurate diagnosis is crucial to preventing further MPP outbreaks during these epidemic cycles. At the onset of MPP, clinical symptoms are often mild, with many children presenting primarily with extrapulmonary symptoms. The disease course is typically prolonged, and despite ongoing clinical efforts, severe extrapulmonary damage can occur. This is often due to the increased invasiveness of the pathogen and subtle pulmonary imaging findings. Commonly affected systems include the nervous, urinary, and coagulation systems, with myocardial and liver cell damage being particularly prevalent (5–7). Research indicates that in patients with severe MPP, myocardial injury can often begin asymptomatically, with 30–40% of patients developing severe cardiovascular complications, such as heart failure, arrhythmias, and cardiogenic shock, which pose serious threats to life (8–11). Additionally, MP accounts for 13% of all pathogenic factors causing hepatocyte damage in children under 12 years (12), posing a serious threat to the life and health of affected children (13, 14). According to previous studies, the main reasons for the progressive aggravation of extrapulmonary damage are the insidious onset of MP infection leading to missed or misdiagnoses, as well as the lack of comprehensive predictive indicators, which can result in extrapulmonary organs progressing from initial biochemical indicator abnormalities and organ involvement to severe extrapulmonary damage. Therefore, how to establish a comprehensive and precise evaluation indicator system during the dynamic evolution of myocardial and liver injuries following MP infection, and to achieve early warning and quantitative monitoring of the progression of extrapulmonary organ damage, has become a key scientific issue that urgently needs to be addressed in the current field of clinical medicine.

Radiomics, as an emerging auxiliary diagnostic technology, has shown advantages in clinical practice. Radiomics transforms quantifiable features of images, such as intensity, shape, and texture, into high dimensional and discoverable digital information that reflects the biological characteristics of lesions. These features have shown particular value in diagnosing lung nodules, tumors, interstitial diseases, and inflammation, as well as in predicting treatment outcomes (15–17). Currently, due to the high incidence of cardiac and hepatic cell involvement following MPP infection and the variability in individual recovery times, there is a lack of effective clinical prediction tools. Therefore, this study aims to construct a predictive model based on radiomics to assess the likelihood of extrapulmonary organ involvement (i.e., myocardial and hepatocyte involvement) associated with MPP infection, and to predict recovery times after treatment. Overall, this model provided a precise prediction tool for the diagnosis and prognosis assessment of MPP-related extrapulmonary organ involvement, with the goal of improving clinical outcomes, reducing the incidence of extrapulmonary complications, and enhancing patient prognosis.

## Materials and methods

### Institutional board approval

This study recruited patients under the age of 14 years, diagnosed with MPP from three medical centers, between December 2022 and December 2024. The data collected included clinical records, laboratory test results, and computed tomography (CT) imaging. Clinical and laboratory data were obtained from electronic medical record systems, while CT imaging data were based on initial admission scans. The investigators were blinded to all patient information, and the study was approved by the Ethics Committee of our medical center (HDFYLL-KY-2024-002), with informed consent being waived.

### Study selection

**Figure 1:**
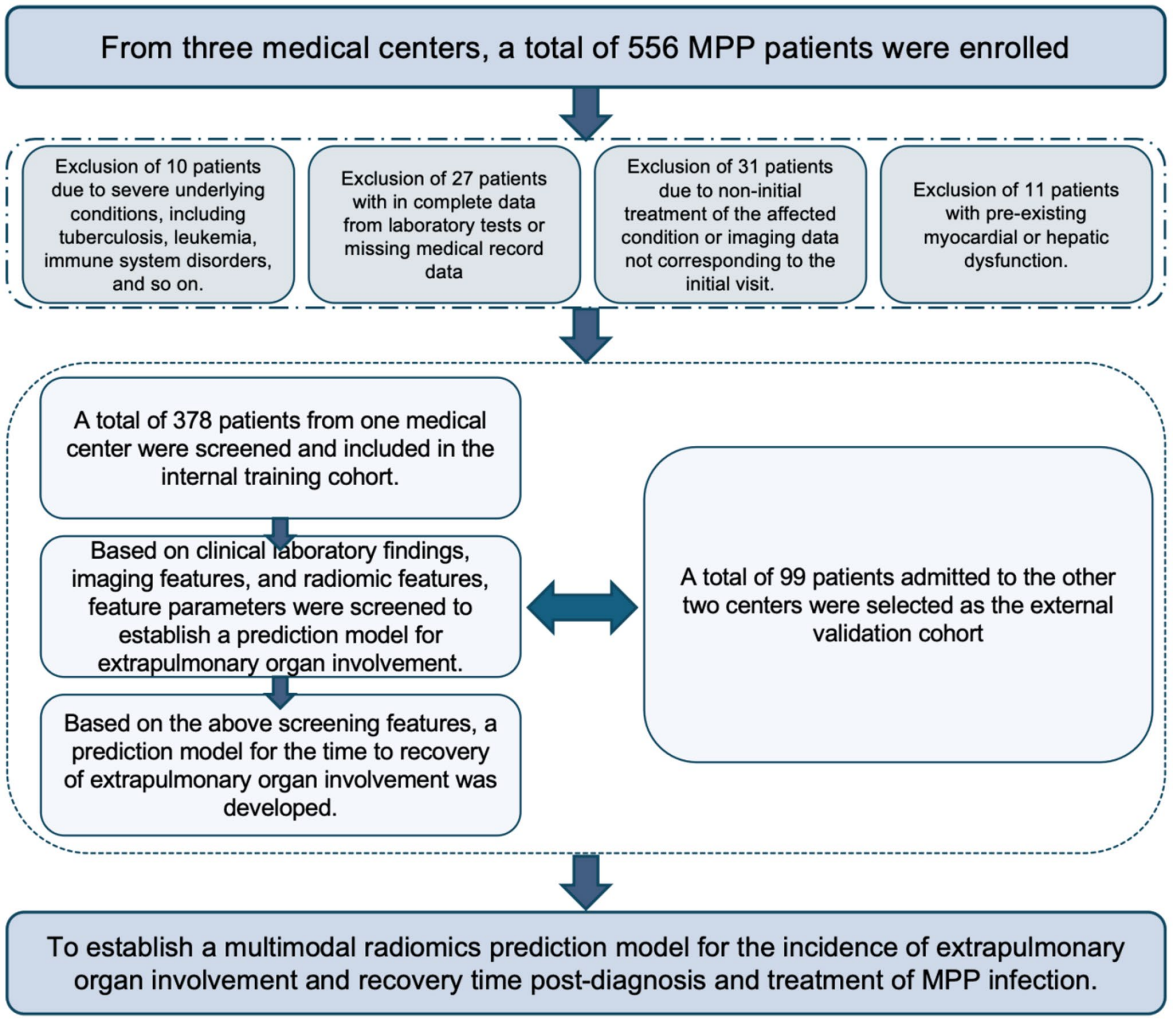
Flowchart of the patient inclusion and exclusion process.

Patients included in the study were rigorously reviewed, based on the diagnostic criteria for *Mycoplasma pneumoniae* pneumonia (MPP) as follows (18, 19): 1. MP antibody titer >1:160 or MP-specific IgM antibody positivity in serum; 2. chest CT showing lobar or lobular patchy infiltration, interstitial lung changes, and hilar lymphadenopathy; and/or, 3. the presence of respiratory symptoms such as fever and cough, with dry or wet rales detectable on lung auscultation. Exclusion criteria were: (1) patients with concurrent diseases such as tuberculosis, lung cancer, hematological disorders, autoimmune diseases, or other major illnesses; (2) patients not undergoing initial treatment or without first-time diagnostic imaging data; (3) patients with a prior history of myocardial or liver cell damage from other causes; and/or (4) incomplete medical records. The patient inclusion and exclusion processes are shown in Figure 1. Regarding criteria for Assessing Extrapulmonary Organ Involvement, Treatment Plan, and Recovery Standards, extrapulmonary organ involvement was defined as Cardiomyocyte involvement, Hepatocyte involvement, or Combined myocardial and hepatocyte involvement. Cardiomyocyte involvement was identified when CK-MB > 25.0 U/L or CK > 200 ng/mL, and Hepatocyte involvement was identified when ALT >30 U/L or AST > 44 U/L. Regarding the treatment plan, following established guidelines, a standard symptomatic treatment approach was used. Regarding recovery standards (18), recovery from extrapulmonary organ involvement was determined by the normalization of laboratory indicators (CK-MB < 25 U/L and CK < 200 ng/mL, ALT <30 U/L and AST < 44 U/L). Once these values returned to normal, treatment was terminated, and the time from the start of treatment to recovery was recorded.

### Clinical and laboratory characteristics

Data collection included general patient information such as sex, age, time to recovery from extrapulmonary organ involvement, duration of fever, and highest recorded body temperature. Laboratory parameters included white blood cell count, neutrophil count, lymphocyte count, monocyte count, eosinophil/basophil count, platelet count, creatine kinase-MB (CK- MB), alaninetransaminase (ALT), aspartate transaminase (AST), lactate dehydrogenase (LDH), activated partial thromboplastin time (APTT), fibrinogen, procalcitonin (PCT), D-dimer, C-reactive protein (CRP), antibody titer, and the systemic immune-inflammation index (SII, platelet count × neutrophil count/lymphocyte count). Additionally, mycoplasma antibody titer and MP-specific antibody positivity were also assessed.

### Image processing and feature extraction

#### Image processing

All patients underwent chest CT scans (detailed scanning parameters are provided in Supplementary Information 1.1). During the scans, patients were in a supine position and trained to hold their breath prior to the scan. The scanning range extended from the lung apex to the base. The images were imported into the DICOM format into the uAI ResearchPortal V1.1 (United Imaging Intelligence, Shanghai, China) for processing. One radiologist with over 10 years of experience in thoracic imaging independently delineated the pulmonary lesions. A second radiologist reviewed the regions of interest (ROI). If there was disagreement, a third radiologist intervened, and the lesion was segmented based on the consensus of all three radiologists, who were blinded to the patients’ clinical information.

#### CT image feature extraction

Two radiologists, each with more than 10 years of experience, independently summarized the CT features, which included the number and size of nodules, the extent of ground glass opacity, pleural effusion, consolidation, “crazy paving” pattern, pleural abnormalities, bronchial thickening, bronchial air signs, bronchial wall thickening, and pneumonia-related lung abnormalities in both lungs. In cases of disagreement, a third senior radiologist reviewed the findings. Again, all radiologists were blinded to clinical data.

#### Radiomics feature extraction

**Figure 2:**
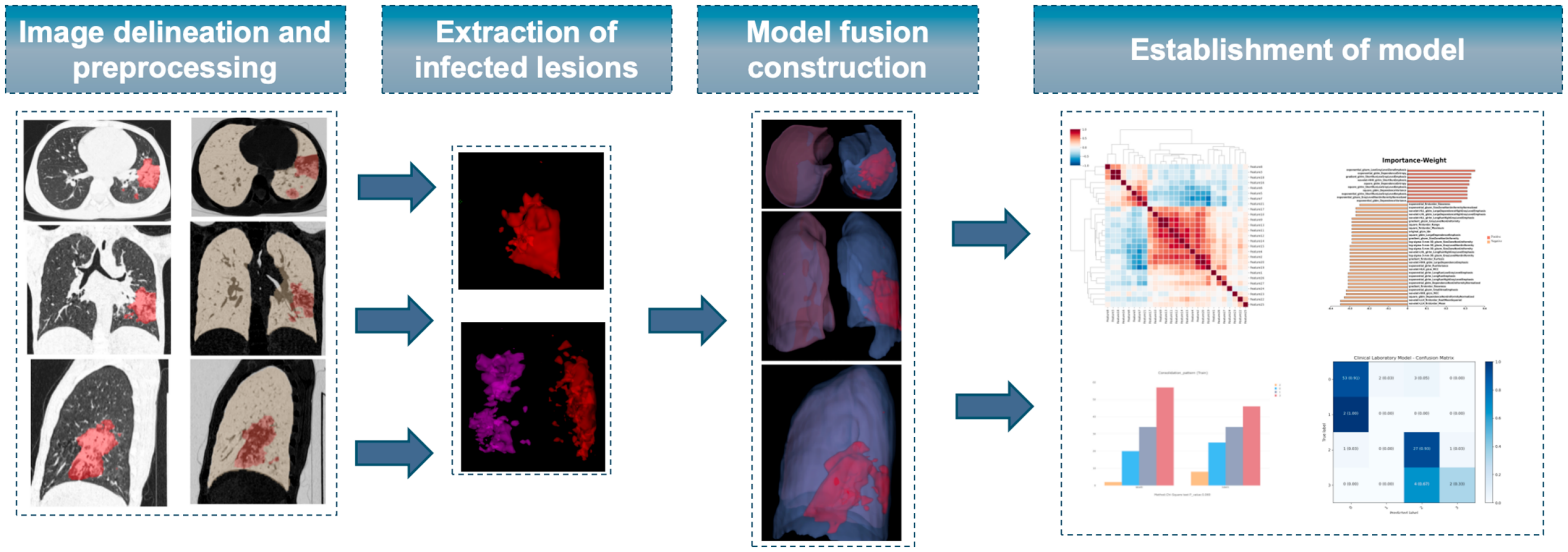
Extraction of image features and establishment of radiomics model.

Radiomics features were extracted using Z-score normalization, wavelet transformation, and Gaussian filters, including the Laplacian of Gaussian operator to preprocess the images and delineated ROIs. Feature extraction was performed using the Pyradiomics open-source package (Python 3.7, version 3.0).^1^ The features were grouped into three main categories: shape, texture, and intensity features. These features were extracted from the original images and further processed using multiple filters to enhance texture and intensity feature extraction. The specific features included the following. 1. Shape features comprising 14 three-dimensional metrics that described the region’s shape and size, such as maximum diameter, volume, surface area, and sphericity. 2. Intensity features comprising 18 features that quantitatively described the distribution of voxel intensities, including mean, peak, maximum, and minimum values. 3. Texture features comprising 72 features that reflected the distribution of pixel intensities and their spatial relationships. These were calculated based on various matrices, including the Gray Level Co-occurrence Matrix (GLCM), Gray Level Run Length Matrix (GLRLM), Gray Level Size Zone Matrix (GLSZM), Neighboring Gray Tone Difference Matrix (NGTDM), and Gray Level Dependence Matrix (GLDM). Fifteen different filters were applied to the images, including BoxMean, Additive Gaussian Noise, Binomial Blur Image, Curvature Flow, Box sigma Image, LoG, Wavelet, Normalize, Laplacian Sharpening, Discrete Gaussian, Mean, Speckle Noise, Recursive Gaussian, and ShotNoise (Figure 2).

#### Feature selection

First, to assess “extrapulmonary organ involvement,” clinical laboratory data, imaging features, and radiomic features were initially screened using an independent samples *t*-test. This test was used to evaluate mean differences between groups with different labels, and Levene’s test was used to assess the homogeneities of variances, ensuring that the selected features were statistically significant. Next, Least Absolute Shrinkage and Selection Operator (LASSO) regression was used for further selection, using its L1 regularization constraint to shrink the regression coefficients of less important features to zero, thereby retaining only the most critical and distinguishing features. A subset of features showing statistical significance (*p* < 0.05) in the univariate analysis was subsequently entered into the LASSO model. The retained features from LASSO were then weighted based on their resulting coefficients. (as shown in Supplementary Information 1.2).

### Construction and evaluation of extrapulmonary organ involvement assessment models

We developed models based on the Support Vector Machine algorithm, incorporating weighted features to create clinical laboratory models, radiomics models, imaging feature models, and a hybrid model. All features were initially standardized using a Standard Scaler to ensure uniformity in the input metrics. The feature selection mechanism from the random forest was used to further refine the feature set. To mitigate the risk of overfitting during model construction, we adjusted the regularization parameter C and kernel coefficient gamma within the SVM framework to control model complexity. To assess model robustness, we used the Bootstrap method, resampling from the original training and validation datasets, and after 1,000 iterations, we calculated the area under the curve (AUC) to evaluate model performances. A 5-fold cross-validation was used to ensure the model’s robustness. Model parameter settings included a variance threshold of 0.01, a random seed of 1, and a maximum depth of 200 for decision trees, validated with both the Training and Testing cohorts.

### Construction and evaluation of recovery time models

Using features from the “Extrapulmonary Organ Involvement Assessment,” we constructed a predictive regression model with the random forest algorithm. The model aimed to predict “Extrapulmonary Organ Involvement Recovery Time” by calculating the Mean Squared Error (MSE), Mean Absolute Error (MAE), and R^2^ score for “Actual Recovery Time” vs. “Predicted Recovery Time.” Data processing and model training were conducted using Python 3.7 and the scikit learn library. A 5-fold cross-validation was used to ensure the robustness of the model evaluation and to prevent overfitting. The random forest model parameters included 300 decision trees, a minimum of 10 samples per node, and a maximum depth of 10 for decision trees, ensuring sufficient model complexity to capture nonlinear relationships in the data. Predictions were expressed in days, with rounding-up to the nearest whole number when the result was not an integer.

### Statistical analysis

This study used Python software (version 3.7).^2^ Quantitative data were represented as medians (Q1–Q3). Data conforming to a normal distribution were analyzed using the Student’s *t*-test, while non-normally distributed data were analyzed using the Mann–Whitney U test. Qualitative data were analyzed using the χ^2^ test, and categorical variables were expressed as frequencies and percentages. A *p*-value of less than 0.05 was considered statistically significant. For the Extrapulmonary Organ Involvement Assessment Model, we used the “scikit learn” package to plot the receiver operating characteristic (ROC) curve, calculate the AUC, calibrate the classifier, and demonstrate the model’s discriminative power across four categories. Decision curve analysis was performed using “dca,” and “scipy.stats” was used for Delong’s test. The Extrapulmonary Organ Involvement Recovery Time model was evaluated using the MSE, MAE, and Modified R^2^ score.

## Results

### Patient characteristics

In this study, we identified 556 patients under the age of 14 years with MPP, from three medical centers based on diagnostic or biochemical results. After applying exclusion criteria, we excluded 10 cases with underlying diseases (such as immune system disorders, leukemia, etc.), 27 cases with missing clinical data or laboratory tests, 31 cases with non-first treatment or non-first chest imaging data, and 11 cases with myocardial or liver function abnormalities not due to MPP, resulting in a final selection of 477 MPP patients. Among them, the Training cohort consisted of 378 patients (male/female: 202/176, average age 7 years), and the Testing cohort consisted of 99 patients (male/female: 50/49, average age 7 years).

### Feature selection

**Figure 3:**
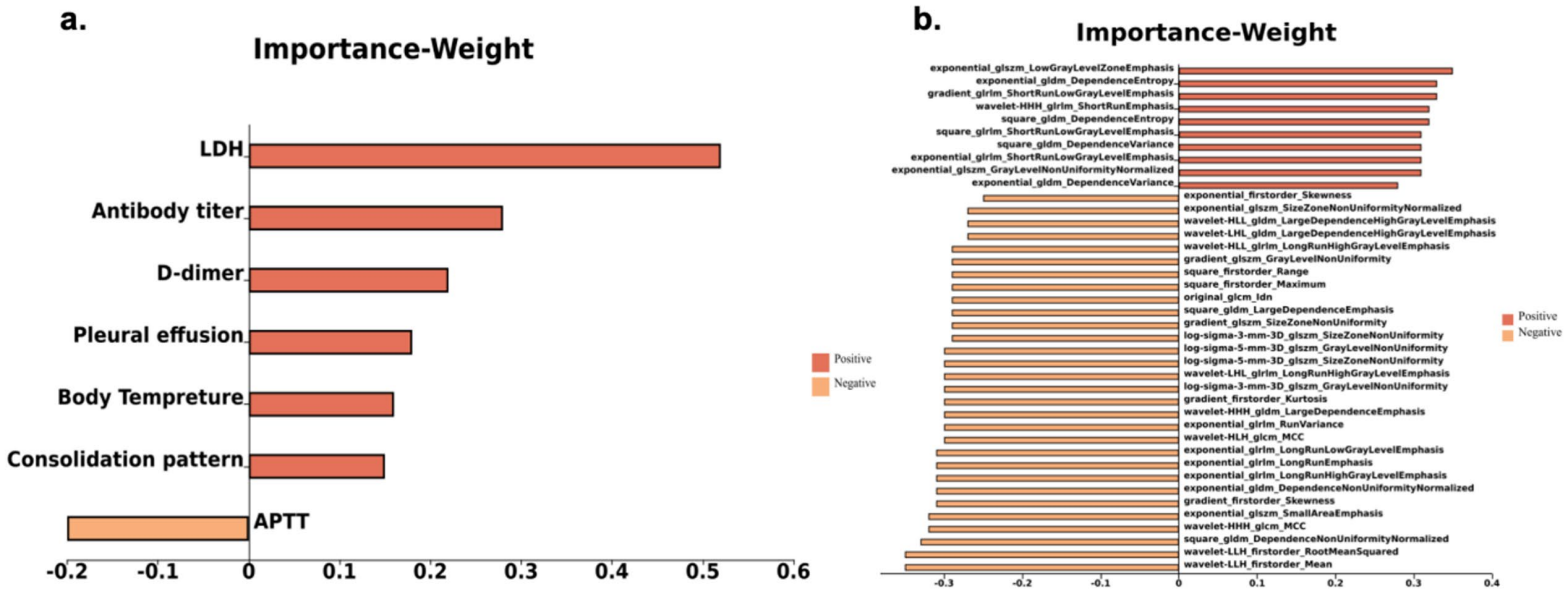
Feature parameter selection and weight values: **(a)** clinical, laboratory, and imaging parameters; **(b)** radiomic features.

Upon analysis, significant differences were observed in the clinical laboratory features of body temperature, LDH levels, D-dimer levels, and antibody titer between the Training and Testing cohorts (*p* < 0.05). APTT was not significantly different in the Training cohort, and PCT differed in the Testing cohort (*p* < 0.05). No significant difference was found in other clinical characteristics or laboratory parameters within or between groups (as shown in Table 1 and Supplementary Tables 1, 4). In CT imaging features, the consolidation pattern showed a clear difference between the Training and Testing cohorts (*p* < 0.05), and pleural effusion was significantly different in the Training cohort (*p* < 0.05). No significant difference was observed in other imaging features within or between groups (as shown in Supplementary Table 2). Using the radiomics features, a total of 40 valuable features were selected, including seven First-order features, 10 GLSZM features, 10 GLDM features, 10 GLRLM features, and three GLCM features. The feature weights and statistical differences are shown in Figure 3 and Supplementary Table 3.

**Table 1:** The characteristics of basic information, laboratory parameters, and imaging features of the patients.

Characteristics	Training cohort (*n* = 378)					Testing cohort (*n* = 99)					*p*-value
	Label = 0	Label = 1	Label = 2	Label = 3	*p*-value	Label = 0	Label = 1	Label = 2	Label = 3	*p*-value	
Basic information											
Gender (Male, %)	145 (54.5)	8 (50.0)	40 (48.7)	9 (60.0)	0.145	24 (42.1)	4 (66.6)	19 (65.5)	3 (42.8)	0.145	0.620
Age	7.0 [5.0, 9.0]	8.0 [6.0, 9.0]	7.0 [5.2, 9.0]	7.0 [5.0, 9.0]	0.901	7.0 [5.0, 8.0]	8.0 [7.2, 8.7]	6.0 [5.0, 8.0]	6.0 [6.0, 8.0]	0.734	0.374
Body Tempreture	39.1 [38.8, 39.6]	39.1 [38.8, 39.4]	39.5 [38.7.40.0]	40.0 [39.3, 40.0]	**0.008**	39.0 [38.5, 39.6]	38.9 [38.5, 39.1]	39.8 [39.1, 40.0]	40.2 [39.5, 40.6]	**0.003**	0.243
Laboratory parameters											
WBC	8.09 [6.30, 10.36]	7.23 [5.80, 8.32]	7.68 [5.70, 10.06]	8.44 [5.96, 9.06]	0.199	7.80 [5.92, 9.40]	7.10 [5.69, 9.60]	8.83 [6.45, 11.81]	9.97 [9.23, 11.83]	0.090	0.714
LDH	264.0 [227.0, 309.7]	304.5 [283.7, 322.2]	367.0 [289.5, 482.2]	487.0 [349.0, 606.5]	**<0.001**	264.0 [217.0, 302.0]	272.0 [238.7, 306.7]	420.0 [334.0, 504.0]	506.0 [351.5, 556.0]	**<0.001**	0.446
APTT	33.1 [31.0, 35.4]	33.2 [31.3, 35.8]	31.6 [29.9, 34.3]	29.8 [27.5, 30.8]	**<0.001**	33.0 [30.8, 34.9]	31.3 [31.1.32.6]	32.4 [29.6, 34.0]	31.1 [28.5, 34.7]	0.409	0.383
PCT	0.10 [0.08, 0.18]	0.12 [0.10, 0.20]	0.11 [0.09, 0.19]	0.17 [0.09, 0.30]	0.252	0.10 [0.06, 0.16]	0.10 [0.07, 0.10]	0.12 [0.09, 0.16]	0.27 [0.22, 0.76]	**0.031**	0.463
D-dimer	217.0 [140.0, 346.7]	230.5 [154.2, 365.2]	408.0 [195.5, 899.0]	736.0 [329.5, 2174.0]	**<0.001**	223.0 [141.0, 321.0]	149.0 [137.0, 239.7]	449.0 [137.0, 2155.0]	1314.0 [989.0, 1803.0]	**0.001**	0.861
Antibody titer	320 [80,640]	320 [160,640]	640 [160,640]	320 [320,1,200]	**0.001**	160 [80,640]	160 [80,160]	640 [320,640]	640 [200,1,200]	**0.017**	0.133
Imaging features											
Pleural effusion (n, %)											
No	254 (95.48)	14 (87.50)	70 (85.36)	11 (73.33)	**0.015**	56 (98.24)	6 (100.00)	26 (89.65)	5 (71.42)	0.266	0.875
Small	7 (2.63)	2(12.50)	10 (12.19)	4 (26.66)		1 (1.75)	0 (0.00)	2 (6.89)	2 (28.57)		
Moderate	3 (1.12)	0(0.00)	1 (1.22)	0 (0.00)		0 (0.00)	0 (0.00)	0 (0.00)	0 (0.00)		
Large	2 (0.75)	0 (0.00)	1 (1.22)	0 (0.00)		0 (0.00)	0 (0.00)	1 (3.44)	0 (0.00)		
Pulmonary consolidation (n, %)											
No	53 (19.92)	1 (6.25)	7 (8.53)	2 (13.33)	**0.013**	12 (21.05)	0 (0.00)	3 (10.34)	1 (14.28)	**0.012**	0.370
Patchy	57 (21.42)	7 (43.75)	16 (19.51)	1 (6.66)		17 (29.82)	2 (33.33)	8 (27.58)	2 (28.57)		
Segmental	76 (28.57)	7 (43.75)	17 (20.73)	3 (20.00)		15 (26.31)	4 (66.66)	5 (17.24)	2 (28.57)		
Wedge-shaped	80 (30.07)	1 (6.25)	42 (51.22)	9 (60.00)		13 (22.80)	0 (0.000)	13 (44.82)	2 (28.57)		

WBC, white blood cell; LDH, Lactic dehydrogenase; APTT, activated partial thromboplastin time; PCT, Procalcitonin. Label 0, No extrapulmonary involvement; Label 1, Cardiomyocyte involvement; Label 2, Hepatocyte involvement; Label 3, Combined myocardial and hepatocyte involvement. All counting types of treatments were expressed as n (%). All continuous data have been expressed as Median (Q1–Q3). Student’s *t*-test was used when variables were normal distribution, and Mann–Whitney U test was used when variables were non-normal distribution for continuous variables and chi-square test was used for qualitative variables to analyze whether there were statistically significant differences. All statistical tests were two-sided, and *p*-value lower than 0.05 were considered statistically significant.

### Extrapulmonary organ involvement assessment model performance analysis

**Figure 4:**
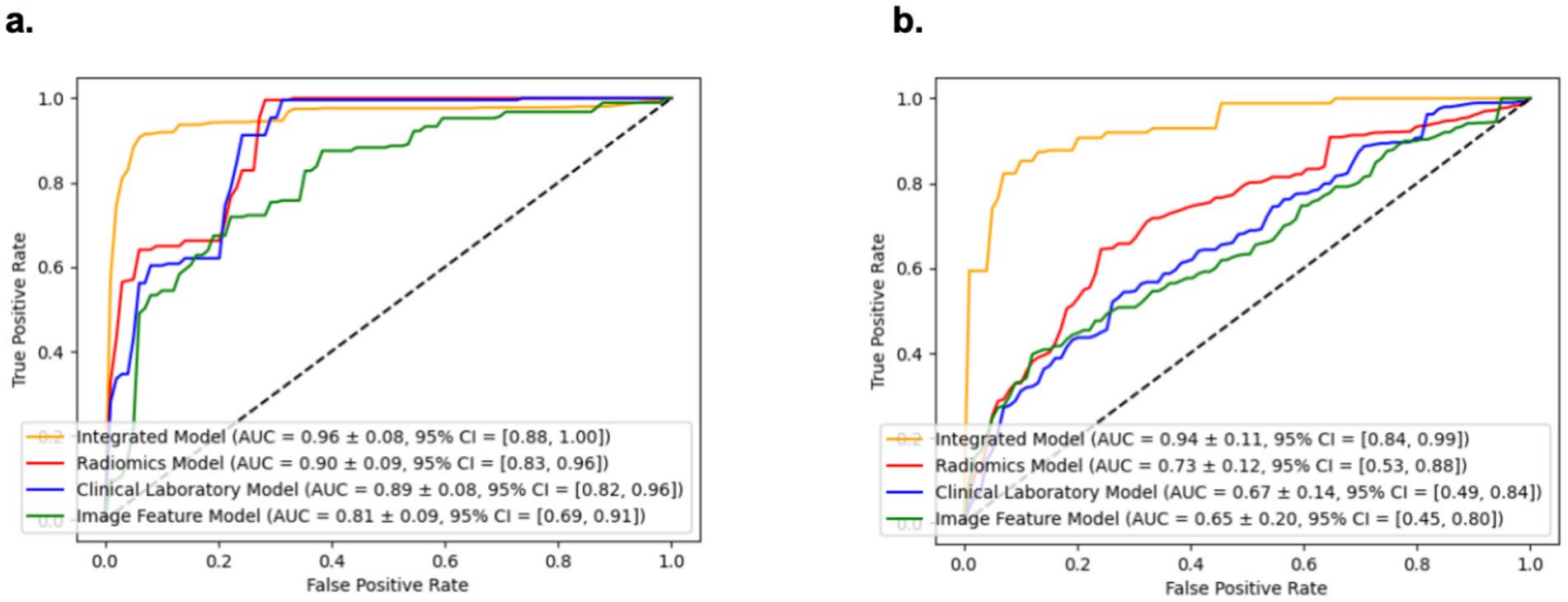
Receiver operating characteristic curve **(a,b)** of the four models in the training cohort and testing cohort.

**Figure 5:**
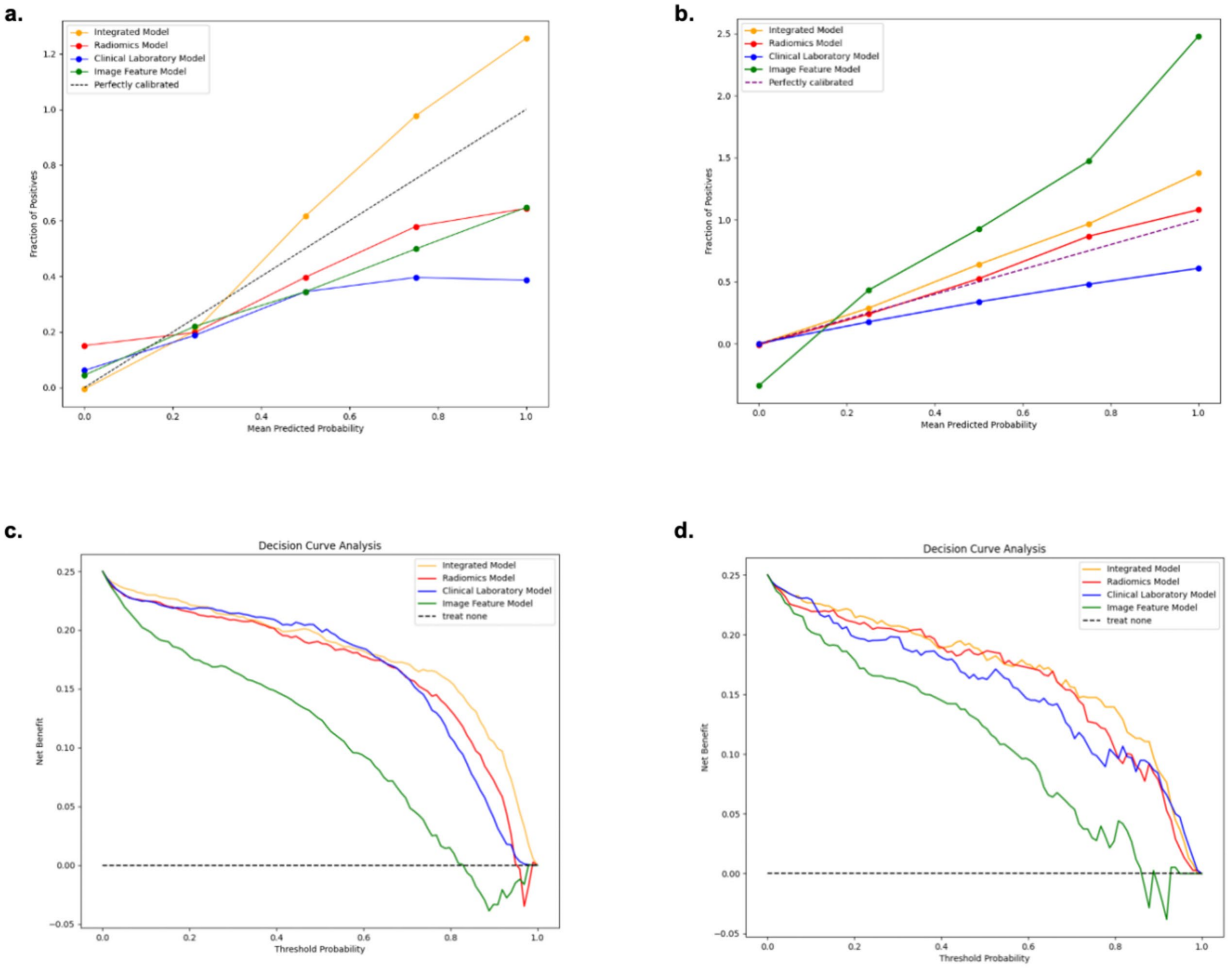
Calibration curve **(a,b)** and decision curve analysis **(c,d)** of the four models in the training cohort and testing cohort.

The performance of the models in distinguishing extrapulmonary organ involvement in MPP patients from the Training and Testing cohorts is shown in Table 2. The ROC curve analysis, calibration curves, and decision curves for the four models are shown in Figures 4, 5. According to the Delong test for model discrimination efficacy, in the Testing cohort, the Radiomics Model showed significant statistical differences when compared with the Clinical Laboratory Model [(AUC = 0.73; 95% CI, 0.53–0.88) vs. (AUC = 0.67; 95% CI, 0.49–0.84), *p* < 0.05]; Image Feature Model [(AUC = 0.73; 95% CI, 0.53–0.88) vs. (AUC = 0.65; 95% CI, 0.45–0.80), *p* < 0.05]. The Clinical Laboratory Model and the Image Feature Model showed significant differences in the combined injury group (*p* < 0.05), but showed no statistical difference in the other groups (*p* > 0.05). Ultimately, the Integrated Model showed significant statistical differences when compared with the three individual mode models: Radiomics [(AUC = 0.94; 95% CI, 0.84–0.99) vs. (AUC = 0.73; 95% CI, 0.53–0.88), *p* < 0.05], Clinical Laboratory [(AUC = 0.94; 95% CI, 0.84–0.99) vs. (AUC = 0.67; 95% CI, 0.49–0.84), *p* < 0.05], and Image Feature Models [(AUC = 0.94; 95% CI, 0.84–0.99) vs. (AUC = 0.65; 95% CI, 0.45–0.80), *p* < 0.05]. indicating that the integrated model had the best predictive performance among all models (Table 3).

**Table 2:** Effectiveness parameters and analysis among different models.

Characteristics	AUC (95% CI)	Accuracy	Precision	Recall	F1-score
Integrated model					
Training cohort	0.96 (0.88,1.00)	0.90	0.88	0.91	0.88
Testing cohort	0.94 (0.84,0.99)	0.86	0.82	0.86	0.84
Radiomics model					
Training cohort	0.90 (0.82,0.96)	0.87	0.81	0.83	0.80
Testing cohort	0.73 (0.53,0.88)	0.84	0.76	0.84	0.80
Clinical laboratory model					
Training cohort	0.89 (0.82,0.96)	0.88	0.80	0.82	0.79
Testing cohort	0.67 (0.49,0.84)	0.83	0.80	0.83	0.80
Imaging feature model					
Training cohort	0.81 (0.69,0.91)	0.79	0.74	0.75	0.70
Testing cohort	0.65 (0.45,0.80)	0.76	0.67	0.76	0.70

AUC, area under curve.

**Table 3:** Delong test of variability of different models in the external testing cohorts.

Characteristics	Label 0		Label 1		Label 2		Label 3	
	z score	*p*-value	z score	*p*-value	z score	*p*-value	z score	*p*-value
Integrated model vs. radiomics model	23.4	**<0.01**	11.9	**<0.01**	23.5	**<0.01**	13.7	**<0.01**
Integrated model vs. clinical laboratory model	28.9	**<0.01**	35.4	**<0.01**	34.5	**<0.01**	34.5	**<0.01**
Integrated model vs. Image feature model	28.5	**<0.01**	35.6	**<0.01**	34.3	**<0.01**	34.9	**<0.01**
Radiomics model vs. clinical laboratory model	19.3	**<0.01**	10.1	**<0.01**	17.7	**<0.01**	10.1	**<0.01**
Radiomics model vs. image feature model	20.4	**<0.01**	13.1	**<0.01**	18.1	**<0.01**	11.1	**<0.05**
Clinical laboratory model vs. image feature model	0.64	=0.51	0.21	=0.83	−1.91	=0.06	2.27	**<0.05**

Label 0, No extrapulmonary involvement; Label 1, Cardiomyocyte involvement; Label 2, Hepatocyte involvement; Label 3, Combined myocardial and hepatocyte involvement.

### Performance analysis of the extrapulmonary organ involvement time prediction model

**Figure 6:**
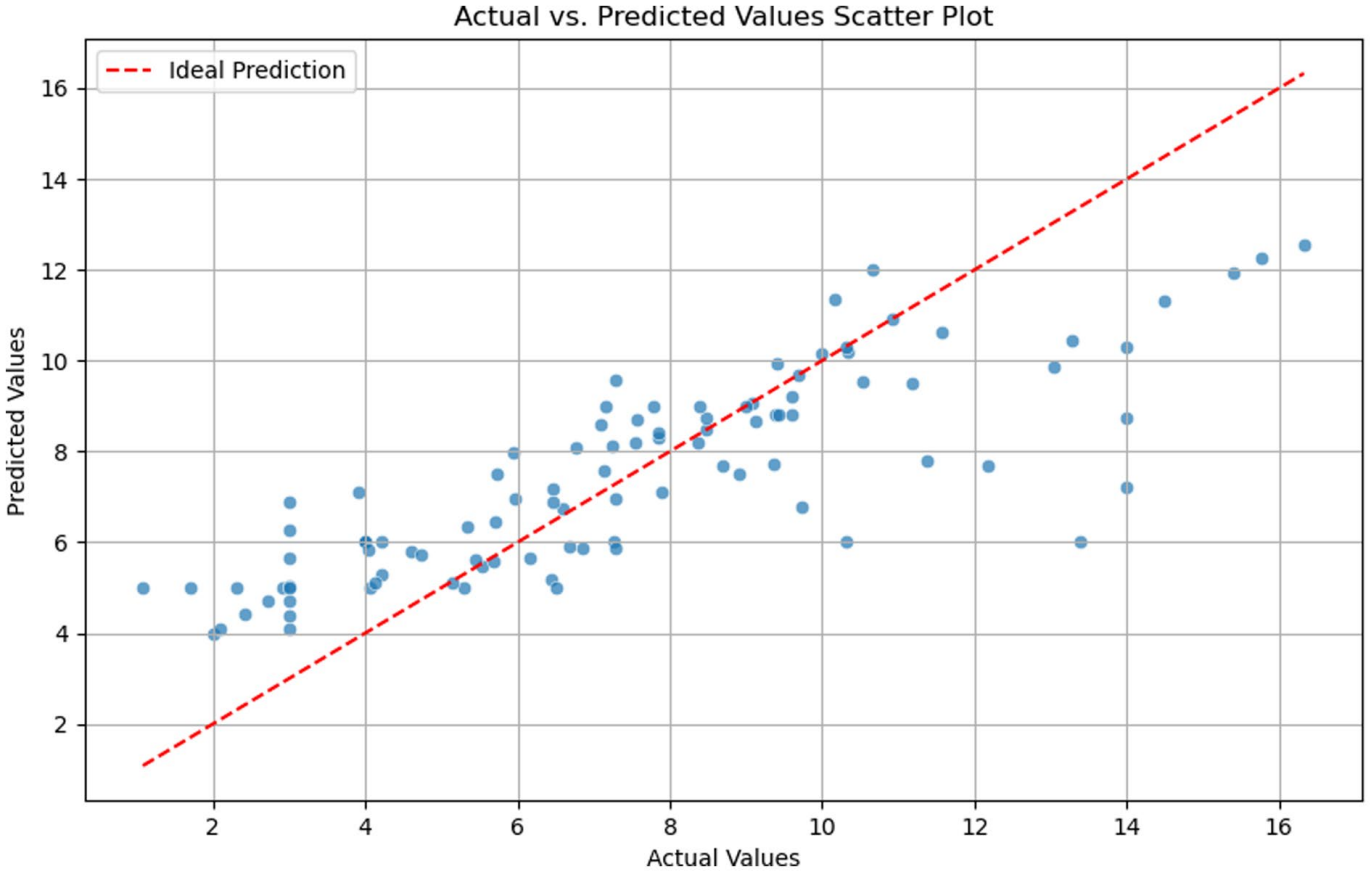
The distribution characteristics of predicted days and actual days in scatter plot.

The model predicted the recovery time for extrapulmonary organ involvement. The Modified Mean Squared Error (MSE) of the model was 6.0 days, indicating that the average squared error of the recovery time prediction from the actual value was 6.0 days. The Modified Mean Absolute Error (MAE) was 1.9, suggesting that the predicted recovery time deviated from the actual value by an average of approximately 1.9 days. The Modified R^2^ Score was 0.6825, which meant that the model accounted for about 68.25% of the data variability, indicating that the model had good predictive accuracy. The scatter plot, based on the statistical process, is depicted in Figure 6.

## Discussion

In this study, we developed models for assessing the probabilities of extrapulmonary organ involvement following MPP infection, and for predicting the recovery times using radiomics technology. We found that the Integrated Model showed the highest efficacy in assessing extrapulmonary injury (AUC of 0.94, accuracy of 0.88, precision of 0.67, recall of 0.86; f1-score of 0.84), and the prediction results for the recovery time of extrapulmonary injury were satisfactory (modified MSE of 6.0, modified MAE of 1.9, and modified R^2^ Score of 0.6825). These findings showed that radiomics-based models had a high predictive value for assessing extrapulmonary organ involvement following MPP infection, and exhibited good performance in predicting the recovery times of such involvement.

In the extrapulmonary organ involvement Assessment Model, clinical laboratory parameters such as LDH, D-dimer, antibody titers, body tempreture and APTT made significant contributions. LDH, an important enzyme in glycolysis, is widely present in vital organs like the lungs and liver (21). Miyashita et al. (22) suggested that LDH is released in large amounts when the body experiences inflammation, tissue damage, or necrosis, correlating positively with the degree of injury. In this study, patients with extrapulmonary organ involvement had higher specificity and sensitivity of LDH, when compared with other MPP patients, consistent with previous findings (23). D-dimer, a degradation product of cross-linked fibrin, indicates the presence of thrombosis and fibrinolysis in the blood (24, 25). Elevated levels of D-dimer, as noted in previous studies, suggest an increased likelihood of myocardial and liver involvement, playing a crucial role in predicting extrapulmonary organ involvement in MPP. Antibody titers, which have been associated with disease severity and the extent of extrapulmonary injury in prior research reports (14, 26), aligned with the weighted features selected in this study and served as an important basis for predicting extrapulmonary organ involvement. Fever is common in MPP patients, in this study, body temperature, a key clinical parameter, may be closely tied to predicting extrapulmonary organ involvement. Prior studies have shown that body temperature is an important factor in predicting the progression of MPP, which aligns with the findings of this study (14, 26). Finally, APTT, the only negatively correlated weighted feature, has not been clearly linked to the severity of pneumonia in previous studies (24, 27). The results of this study may be related to sample size and patient demographics, suggesting that the relationship between APTT and the severity of pneumonia may require further investigation.

Regarding imaging features, pleural effusion and Pulmonary consolidation were identified as a dominant feature in constructing the imaging feature model. Previous studies have shown that acute infections like MPP can lead to oxidative stress reactions (28–30), after adherence to respiratory epithelial cells, further causing pleural effusion, which can more fully reflect the severity and prognosis level of MPP. In this study, most patients with extrapulmonary injury had pleural effusion, consistent with previous research (22, 31). Pulmonary consolidation arises from alveolar cell - induced immune responses, causing prominent plasmacytic inflammation around bronchi and vessels. Strong inflammation leads to excessive alveolar exudate accumulation, causing lung consolidation, which often signifies severe illness and aligns with prior studies (22, 31). However, due to subtle differences in extrapulmonary injury imaging, even experienced radiologists may not discern significant differences, resulting in the imaging feature model consistently performing at the lowest efficacy, when compared with similar models used in both the Training and Testing cohort.

Radiomics features, obtained from quantitative information in imaging data, use texture features to reflect the spatial relationships between voxels and high order statistical measures, to reveal the specificity of different lesions. The radiomics information of the ROI is extracted, resulting in a special imaging pattern similar to a clinical diagnosis (32). In the present study, out of 1,904 calculated radiomics features, 41 dominant features were ultimately retained. The analysis results showed that the radiomics model, as a single model, had the highest predictive efficacy, with AUC values of 0.90 for the internal training set and 0.73 for the external test set. We believe that radiomics, by extracting subtle imaging signs that are difficult for the naked eye to discern, compensated for the omission of key features due to human factors, thereby improving diagnostic efficacy. Although the basic diseases in different groups included in this study were similar, the lung features after extrapulmonary organ involvement still showed a certain degree of heterogeneity. For example, matrix texture changes such as GLSZM and GLRLM were particularly sensitive, which may have had a certain degree of specificity in distinguishing different manifestations caused by the same pathogenic mechanism, when compared to simple imaging features, and may be the key to distinguishing the presence or absence of extrapulmonary organ involvement.

The integrated model, based on feature weights, demonstrated excellent performance in assessing extrapulmonary injury, with AUC values reaching 0.96 in the training cohort and 0.94 in the testing cohort. We believe that the addition of radiomics significantly enhanced the assessment capabilities of clinical laboratory tests and CT imaging features. Although no similar studies on the assessment of extrapulmonary organ involvement following MPP infection have been reported, the addition of radiomics has proven to significantly enhance the predictive efficacy of models in studies of refractory MPP, COVID-19, and other pulmonary infectious diseases (33, 34), which is consistent with our research findings.

In predicting the recovery times after treatment, we used features selected from the extrapulmonary organ involvement model and constructed a predictive regression model using the random forest. By comparing the “actual recovery time” with the “predicted recovery time,” the Modified MSE was 6.0, the Modified MAE was 1.9, and the Modified R^2^ Score was 0.6825. These results indicated that our model had high predictive accuracy and goodness of fit. The low values of Modified MSE and Modified MAE indicated that the model’s prediction error was small, while the high value of the Modified R^2^ Score indicated that the model could explain the variabilities in the data. We therefore believe that this model adequately predicted recovery times, which is particularly useful for remote areas or grassroots institutions lacking sophisticated healthcare capabilities.

## Limitations

This study had certain limitations. First, although it was a multicenter study involving clinical data, CT imaging, and laboratory indicators, the small sample size may still have introduced bias when studying extrapulmonary organ involvement outcomes. Second, while myocardial and hepatocyte injuries are common complications of MPP, extrapulmonary organ involvement such as those to the hematopoietic, skin, and nervous systems were not included. Therefore, in the future, we hope to expand the sample size, to include more laboratory monitoring indicators, not limited to children, to include more adult patients to more comprehensively predict extrapulmonary organ involvement and recovery times across all age groups.

## Conclusion

This study enhanced the predictive efficacy of clinical laboratory parameters and imaging features for assessments of MPP with extrapulmonary injury and determinations of recovery times of extrapulmonary injury using radiomics. It provided an effective diagnostic and therapeutic tool to improve the diagnostic efficiencies of clinical physicians and reduce the risk of disease progression in patients.

## Data Availability

The original contributions presented in the study are included in the article/Supplementary material, further inquiries can be directed to the corresponding author.

## Footnotes

1
https://pyradiomics.readthedocs.io/en/latest/installation.html

2
https://www.python.org/getit/
