# Supplementary material for: Development of a radiomics-based model using computed tomography imaging to assess the incidence of extrapulmonary organ involvement in Mycoplasma pneumoniae pneumonia and to predict recovery times: a multicenter study
Source: Front Med (Lausanne). 2026 Jan 15;12:1732165. doi: 10.3389/fmed.2025.1732165 (PMC12851944; doi:10.3389/fmed.2025.1732165)
Supplement: Supplementary file 1 [file Supplementary_file_1.docx]

# Appendix

**Figure 1.** *Thematic map of the six analytical themes derived from interview data*


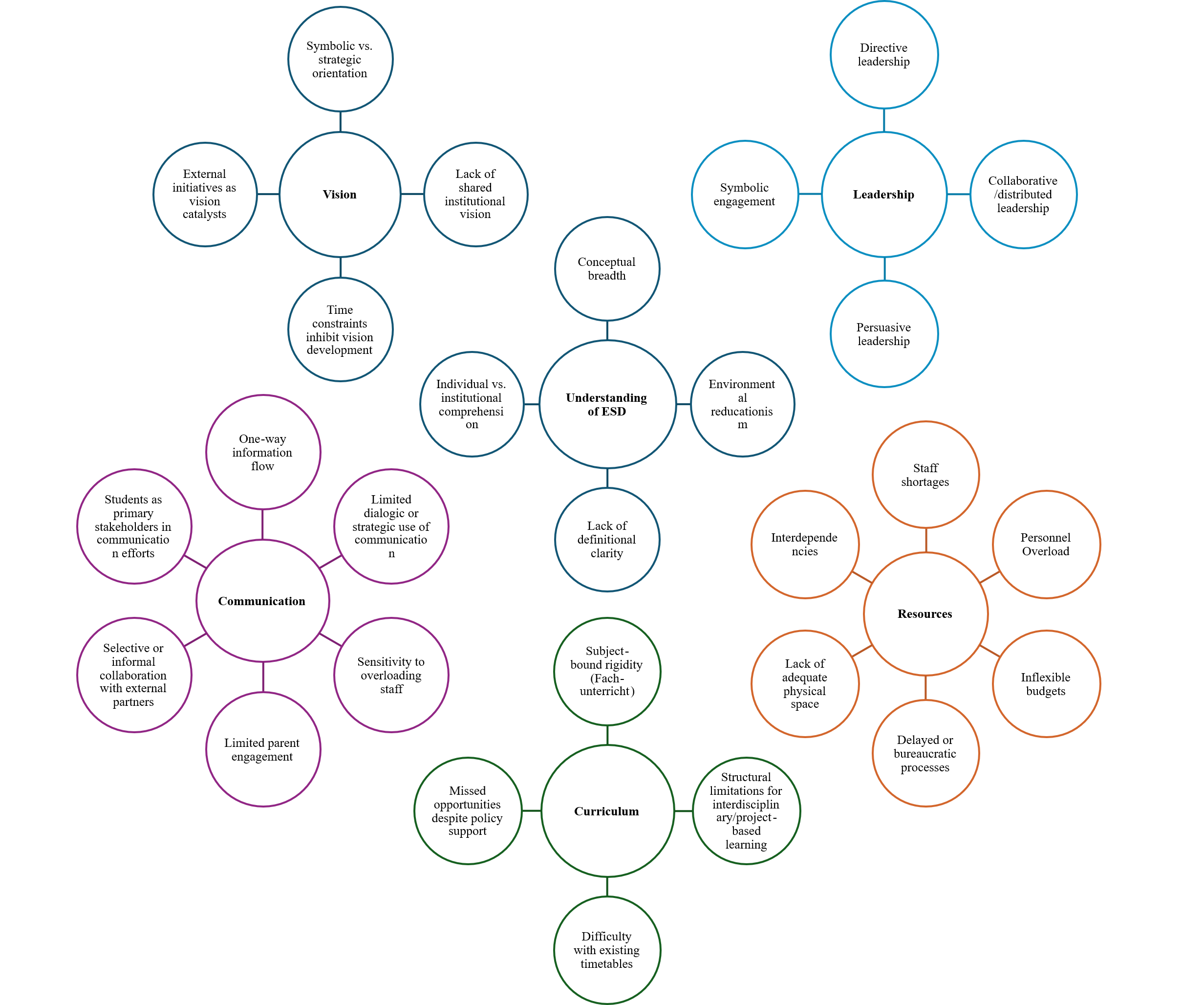


| Sub-theme of analytical theme „Vision“ | Quote (German) | Quote (English translation) | Speaker |
| --- | --- | --- | --- |
| Symbolic vs. strategic orientation | *„Die Vision muss gelebt werden. Es reicht nicht, sie ins Schulprogramm zu schreiben.“* | *“The vision has to be lived. It’s not enough to write it into the school program.”* | S06 |
| Lack of shared institutional vision | *„Unsere Schule steht für Demokratie und Toleranz. […] aber wir haben das nie systematisch [mit Nachhaltigkeit] verbunden.“* | *“Our school stands for democracy and tolerance. […] but we’ve never systematically connected the two [with sustainability].”* | S09 |
| Vision dependent on individual actors | *„Was ist notwendig, ist aus meiner Sicht die Erarbeitung eines neuen Leitbildes.“* | *“What is necessary, in my view, is to develop a new school mission statement.”* | S03 |
| Time constraints inhibit vision development | *„Ich sehe Nachhaltigkeit als wichtig an, aber ehrlich gesagt fehlt uns die Zeit, daraus eine echte Vision zu entwickeln.“* | *“I see sustainability as important, but to be honest, we lack the time to turn it into a real vision.”* | S10 |
| External initiatives as vision catalysts | *„Ich glaube, dass die Lehrkräfte bei uns auch merken, dass es nicht zielführend ist, dass einer da vorne kommt, 45 Minuten erzählt und die anderen 30 sitzen hören zu, sondern dass ich selber ins reguläre Lernen kommen. Und davon ist einfach ein FREI DAY glaube ich ein gutes Element, um Schule zu verändern.“* | *“I think the teachers here are also starting to realize that it’s not effective for one person to talk for 45 minutes while 30 others just sit and listen – FREI DAY is, I believe, a good element for changing how school works.”* | S01 |

**Table 1.** *Sub-themes and illustrative quotes for the analytical theme "Vision".*
